# Signaling via TLR2 and TLR4 Directly Down-Regulates T Cell Effector Functions: The Regulatory Face of Danger Signals

**DOI:** 10.3389/fimmu.2013.00211

**Published:** 2013-07-25

**Authors:** Alexandra Zanin-Zhorov, Irun R. Cohen

**Affiliations:** ^1^Kadmon Research Institute, New York, NY, USA; ^2^The Department of Immunology, The Weizmann Institute of Science, Rehovot, Israel

**Keywords:** HSP60, TLR2, TLR4, direct signaling, T cell inhibition, inflammation, LPS

## Abstract

Toll-like receptors (TLRs) are widely expressed and play an essential role in the activation of innate immune cells. However, certain TLRs are also expressed on T cells, and TLR ligands can directly modulate T cell functions. Here, we discuss findings indicating that T cells directly respond to Heat Shock Protein (HSP) 60, a self molecule, or to the HSP60-derived peptide, p277, via a TLR2-dependent mechanism. HSP60 has been considered to be a “danger signal” for the immune system because of its ability to induce pro-inflammatory phenotypes in innate immune cells – in this case via TLR4 activation; nevertheless, TLR2 engagement by HSP60 on T cells can lead to resolution of inflammation by up-regulating the suppression function of regulatory T cells and shifting the resulting cytokine secretion balance toward a Th2 phenotype. Moreover, T cell TLR4 engagement by LPS leads to up-regulation of suppressor of cytokine signaling 3 expression and consequently down-regulates T cell chemotaxis. Thus, TLR2 and TLR4 activation can contribute to both induction and termination of effector immune responses by controlling the activities of both innate and adaptive immune cells.

## Toll-Like Receptors Function in Innate and Adaptive Immune Cells

A key issue in immunology is to understand how the immune system is able to discriminate between self and non-self, inhibiting autoimmune responses, but allowing effective immune responses against microbial antigens (1, 2). One of the mechanisms evolved by the immune system is expression of pathogen recognition receptors, such as Toll-like receptors (TLRs) on immune cells that encounter pathogen-associated molecular patterns (PAMPs) (3). TLRs are a highly conserved class of receptors that are involved in regulation of both innate and adaptive immunity. All TLR belong to the type 1 trans-membrane glycoprotein receptor family with molecular weights ranging between 90 and 115 kDa and containing 16–28 extracellular leucine-rich repeat domains (4). The intracellular C-terminal domain is known as the Toll/IL-1 receptor domain, which shows homology with that of the IL-1 receptor. This domain is required for the interaction and recruitment of various adaptor molecules to activate the down-stream signaling pathway, including the transcription factors NF-κB, AP-1, and IRF (5).

Both humans and mice express TLR1-9; in addition humans, but not mice, express TLR10 and mice exclusively express TLR11-13 (6). TLR are expressed in two distinct cellular compartments (7). In humans, TLR1, TLR2, TLR4, TLR5, and TLR6 are located on the outer membrane and recognize mainly bacterial surface-associated PAMPs like peptidoglycan and lipopeptides (TLR1, 2, 6), lipopolysaccharide (TLR4), and flagellin (TLR5). The other human TLRs are expressed on the membrane of intracellular endosomes, where they bind viral dsRNA (TLR3), ssRNA (TLR7 and 8) or unmethylated bacterial DNA (TLR9) (8). Also, as we shall discuss below, endogenous host molecules can also function as TLR ligands.

Toll-like receptors are widely expressed in innate immune cells, such as macrophages, dendritic cells (DCs), but also in non-immune cells, such as endothelial and epithelial cells (3, 6). In DCs, TLR signaling triggers a maturation program that includes up-regulation of MHC and co-stimulatory molecules, and expression of pro-inflammatory cytokines, such as TNF-α, IL-1, and IL-6. This maturation of DCs significantly increases their ability to prime naïve T cells (9).

More recent TLR expression profiling studies have revealed that certain TLRs are expressed not only in innate immune cells but also in various adaptive immune cells, such as B cells (10, 11), CD4^+^ and CD8^+^ (12, 13), γδ T cells (14), and the CD4^+^CD25^+^ regulatory T cell population (15–17); TLR ligands can directly modulate the function of these adaptive immune cells. When TLR4 signaling induces proliferation and cytokine secretion in naïve mouse B cells (10); several natural and synthetic ligands, including bacterial lipopeptides Pam_3_CSK4 (TLR1/TLR2), flagellin (TLR5), and R-848 (TLR7/8) were found to co-stimulate proliferation and cytokine secretion in human memory CD4^+^ T cells (18, 19). In addition, the TLR3 ligand poly(I:C) and TLR2 ligands increase IFN-γ and IL-6 secretion in TCR-stimulated γδ T cells (20, 21). Furthermore, TLR ligands have been reported to promote the survival and modulate the suppressive capacity of regulatory T cells (17, 22, 23). Thus, the involvement of TLR signaling in modulation of immune response is not limited to innate immune cells.

## TLR2 Signaling Mediates the Innate Effects of HSP60 on T Cells

Heat shock proteins (HSP) are highly conserved proteins induced in response to cellular stress, such as heat shock or nutrient deprivation (24, 25), and function as an endogenous danger signal of the immune system. Inside cells, HSP molecules assist the folding of newly synthesized proteins, participate in protein transport across membranes and refold proteins denatured during cell stress (26). However, HSPs, and in particular, HSP60 interests immunologists because, in addition to serving as a chaperone, extracellular HSP60 could directly activate innate immune cells, including macrophages and DCs (27, 28), through binding to various cell-surface receptors such as CD14, CD40, TLRs and the scavenger receptors CD36 and CD91. However, it was argued that many of the reported pro-inflammatory effects that result from exposure of cells to HSP60 are actually mediated through LPS or other microbial compounds contaminating the HSP60 (29, 30). Nevertheless, highly purified HSP60 was shown to be able trigger inflammatory responses *in vivo* via TLR2 and TLR4 signaling (31). Indeed, it is becoming clear that the self-HSP60 molecule and its synthetic peptides are able to activate TLR signaling (32).

In addition to functioning as a danger signal to innate immune cells, HSP60 also functions as an antigen in host defense and signals through “adaptive” immune receptors, such as T and B cell receptors (33, 34). Autoimmunity to self-HSP60, moreover, does not necessarily cause disease. The cord blood of newborn humans, like the peripheral blood of adults, manifests a relatively high frequency of T cells that can recognize HSP60 (35), and healthy adults manifest T cell reactivity to HSP60 (36). In direct contrast to the function of HSP60 as a danger signal and its involvement in autoimmunity, HSP60 and the HSP60-derived peptide p277 were also found to arrest the destructive inflammation responsible for development of autoimmune diseases such as adjuvant arthritis and type 1 diabetes (37, 38). In a double-blind, Phase II clinical trial the administration of p277 after the onset of clinical diabetes preserved the endogenous levels of C-peptide (a marker of insulin-producing capacity of the pancreas) and was associated with lower requirements for exogenous insulin compared with treatment with a placebo, revealing the arrest of inflammatory β-cell destruction. Treatment with p277 was associated with an enhanced Th2 response to HSP60 and p277 (39). Recently, a large multi-center phase III trial of p277 (DiaPep277) has confirmed the finding of the published phase II study (submitted for publication). Taken together, these results suggest that treatment with HSP60 or its p277 peptide can lead to the induction of HSP60-specific regulators, including anti-ergotypic regulators (40) that can control the collective of pathogenic re-activities involved in the progression of autoimmune diabetes.

The fact that both B and T cells also express TLRs on their surface raised the question about the direct function of these receptors in the regulatory effects of HSP60 on adaptive immune cells. Indeed, recent studies in our laboratory demonstrated that TLR2, but not TLR4 is involved in HSP60-mediated inhibition of T cell chemotaxis via up-regulation of the suppressor of cytokine signaling (SOCS)3 transcription factor (41). In addition, both human and mouse T cells treated with soluble HSP60 or HSP60-derived peptide undergo a signal transduction cascade, activate integrin receptors and induce adhesion to fibronectin via TLR2-dependent signaling (12). Since T cell chemotaxis is a highly coordinated process, which includes the rapid and reversible adhesiveness to extracellular matrix, the ability of HSP60 to induce T cell adhesion via TLR2 may partially contribute to inhibition of T cell chemotaxis.

The involvement of TLR2 in direct effects of HSP60 on T cell function was confirmed in additional studies demonstrating that HSP60 modulates the expression of Th1/Th2 transcription factors (42). It was shown that HSP60 down-regulates expression of the Th1-cell-promoting transcription factor T-bet, the transcription factor NF-κB, and the intracellular-signaling molecule NFATp; HSP60, in contrast, up-regulates the expression of the Th2-cell-promoting transcription factor GATA-3. This leads, in turn, to decreased secretion of TNF-α and IFN-γ and enhanced secretion of IL-10 (42). These innate effects of HSP60 were specifically dependent on intact TLR2, but not TLR4 signaling. In contrast, the ability of HSP60 to induce IL-10 and IL-6 secretion in mouse B cells was largely mediated through TLR4 and MyD88 signaling (10). Thus, HSP60 can directly modulate the adaptive immune cell function via TLR2 and TLR4 signaling pathways, although a direct interaction between HSP60 and these TLRs has not yet been shown.

## The Regulatory Face of Danger Signals

The involvement of CD4^+^CD25^+^ regulatory T cells (Tregs) in HSP60-mediated suppression of T cell responses seems to be an attractive explanation for the protective effect of the molecule *in vivo* as was mentioned above. Indeed, the HSP60 molecule can function as a co-stimulator of Tregs by way of an innate signaling pathway that involves TLR2 (17). Treatment of Tregs with HSP60, or its peptide p277 before anti-CD3 activation significantly enhanced the ability of relatively low concentrations of the Tregs to down-regulate CD4^+^CD25^−^ or CD8^+^ target T cells, detected as inhibition of target T cell proliferation and IFN-γ and TNF-α secretion. The enhancing effects of HSP60 co-stimulation on Tregs involved innate signaling via TLR2, led to activation of PKC, PI-3 kinase, and p38, and were further enhanced by inhibiting ERK. HSP60-treated Tregs suppressed target T cells both by cell-to-cell contact and by secretion of TGF-β and IL-10. The down-regulated target T cells manifested inhibited ERK, NF-κB, and T-bet (17). The contribution of TLR2 signaling to the control of Treg suppressive function still remains controversial and various results have been obtained in different species using different ligands. In rabbits, the engagement of TLR2 down-regulates the suppressive ability of Tregs purified from conjunctiva, and leads to the induction of an HSV-specific effector T cell response *in vivo* (43). In mice, the known exogenous agonist of TLR2, Pam_3_Cys was shown to reverse Treg function in two studies (15, 16), but had no effect on Foxp3 expression and suppressive activity in murine Tregs in work reported by Chen et al. (44). Interestingly, all three studies agreed on the ability of Pam_3_Cys to induce proliferation and promote murine Treg survival. Also, we found that relatively lower concentrations of Pam3Cys as well as polysaccharide A (PSA) of *B. fragilis* could augment Treg inhibition of cytokine secretion by CD4^+^CD25^−^ T cells via TLR2 signaling in humans (17) and mice (22). In contrast, higher concentrations of Pam_3_Cys (1 and 5 μg/ml) were reported to down-regulate human Treg function, but controversial data were obtained with regards to the ability of this TLR2 ligand to down-regulate Foxp3 expression in those two studies (45, 46). Although the precise role of TLR2 signaling in controlling Treg activity needs further characterization, HSP60 via TLR2 acts as a co-stimulator of Treg function.

LPS is known to be involved in activation of macrophages and B cells via TLR4-dependent signaling (3, 6). Although TLR4 expression is detected in T cells (12, 17), LPS was reported not to affect T cell cytokine secretion or proliferation or to activate regulatory T cell function (16, 17, 19, 42, 47). However, certain aspects of LPS-mediated effects on cytokine secretion in T cells via TLR4 *in vitro* did not fully correlate with the pattern seen *in vivo*: TLR4 signaling in CD4^+^ T cells was shown to be inhibitory in a spontaneous model of colitis (48), whereas Reynolds et al. demonstrated that TLR4 signaling promoted the development of experimental autoimmune encephalomyelitis (EAE) in mice (49). Interestingly, the TLR-4-dependent inhibition of colitis was primarily mediated through Th1, whereas the promotion of EAE involved mainly the Th17 subset suggesting that further investigation is required to clarify the molecular mechanism of TLR4-mediated regulation of different T cell subsets *in vivo*. Consistent with the proposed anti-inflammatory function of direct TLR4 signaling, we demonstrated that LPS can up-regulate human T cell adherence to fibronectin and down-regulate the ability of T cells to migrate toward CXCL12 by way of STAT3-dependent induction of SOCS3 expression *in vitro* (50). This response to LPS was mediated specifically via TLR4, but not TLR2 signaling and required the presence of functional MyD88 (50). Thus, LPS, through TLR4 signaling can affect directly the pro-inflammatory T cell function and lead to termination of effector immune responses.

## The Bottom Line

**Figure 1:**
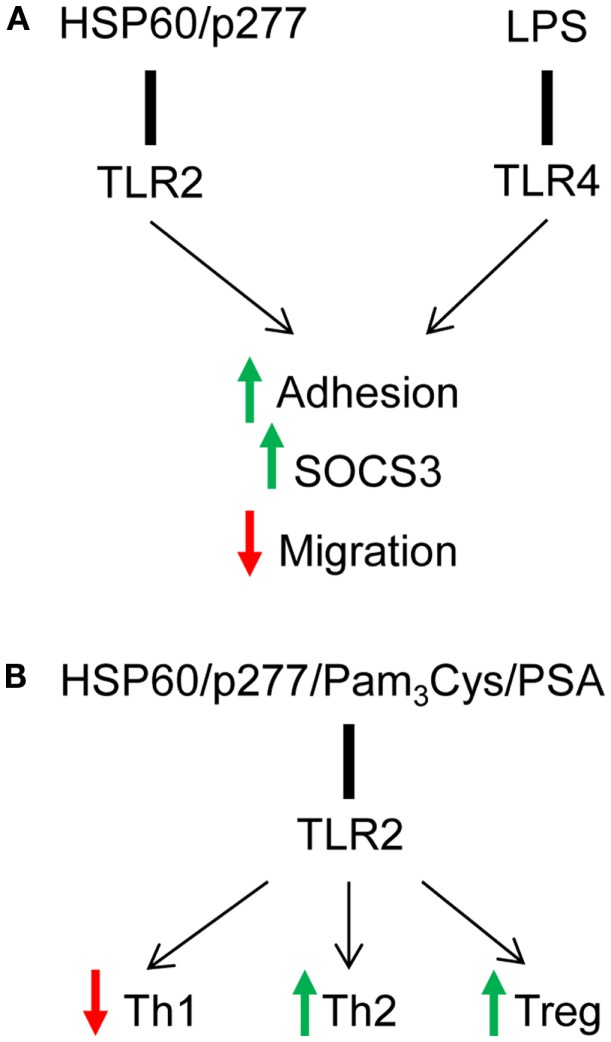
**Signaling via TLR2 and TLR4 directly down-regulates T cell effector function**. **(A)** HSP60 via TLR2 and LPS via TLR4 induce T cell adhesion and down-regulate T cell chemotaxis in SOCS3-dependent mechanism. **(B)** TLR2 signaling induced by several endogenous and pathogen-derived ligands shifts cytokine profile toward Th2-like phenotype and up-regulates the suppressive function of Tregs.

About two decades ago, Janeway hypothesized that regulation of T cells by APCs must be controlled by receptors with specificity for microbial products; indeed, a class of innate receptors restricted to the recognition of non-self antigens was proposed to mediate the ability of the immune system to discriminate between self and pathogens (51). The function of TLR family appeared to fit this hypothesis, and the results of several studies supported the idea that TLRs do play an important role in controlling adaptive immune responses (3). Although TLRs have classically been studied on innate immune cells, recent reports have demonstrated their expression on adaptive immune cells, T and B cells in both mice and humans. Here we have discussed that the endogenous self-protein HSP60 as well as bacterial components, such as LPS directly signal to T cells and induce adhesion, SOCS3 expression that consequently leads to down-regulation of T cell migration via TLR2 and TLR4 respectively (Figure 1A) (12, 41, 50). Interestingly, although LPS-induced signaling through TLR4 had no effect on cytokine secretion in T cells (19, 42, 47), TLR2 signaling induced by both HSP60 and bacterial components, such as Pam_3_Cys and PSA, resulted in the down-regulation of Th1 and up-regulation of Th2-like responses, and the induction of Treg function (Figure 1B) (17, 22, 42). The difference between TLR-mediated effects of HSP60 and LPS on T cells may result from different levels of sensitivity: T cells are extremely sensitive to HSP60 and respond to concentrations in the 0.1–1 ng/ml range (12, 17, 41, 42); the effects of LPS on T cell adhesion and migration require concentrations of about 100 ng/ml (50). Thus, the involvement of different TLRs, TLR2 for HSP60 vs. TLR4 for LPS as well as different degrees of sensitivity contribute to variation between effects of those TLR ligands on T cell function: notably, TLR2 signaling is involved in direct effects of both endogenous signals (HSP60 and HSP60-derived peptide) and pathogen-derived ligands (Pam_3_Cys and PSA). In summary, these findings suggest that direct TLR2 and TLR4 signaling in T cells can modulate decisions dictated by antigen-presenting cells and shift the immune response from a damaging to a healing type.

## Conflict of Interest Statement

The authors declare that the research was conducted in the absence of any commercial or financial relationships that could be construed as a potential conflict of interest.
